# Rapidly Simultaneous Determination of Six Effective Components in *Cistanche tubulosa* by Near Infrared Spectroscopy

**DOI:** 10.3390/molecules22050843

**Published:** 2017-05-19

**Authors:** Xinhong Wang, Xiaoguang Wang, Yuhai Guo

**Affiliations:** College of Agronomy, China Agricultural University, Yuanming Yuan West Road, Haidian District, Beijing 100193, China; wangxinhong120@126.com (X.W.); xgwang@cau.edu.cn (X.W.)

**Keywords:** *Cistanche tubulosa*, high performance liquid chromatography, near infrared spectroscopy, partial least squares

## Abstract

Quantitative determination of multiple effective components in a given plant usually requires a very large amount of authentic natural products. In this study, we proposed a rapid and non-destructive method for the simultaneous determination of echinacoside, verbascoside, mannitol, sucrose, glucose and fructose in *Cistanche tubulosa* by near infrared spectroscopy (NIRS). Near infrared diffuse reflectance spectroscopy (DRS) and high performance liquid chromatography (HPLC) were conducted on 116 batches of *C. tubulosa* samples. The DRS data were processed using standard normal variety (SNV) and multiplicative scatter correction (MSC) methods. Partial least squares regression (PLSR) was utilized to build calibration models for components-of-interest in *C. tubulosa*. All models were then assessed by calculating the root mean square error of calibration (RMSEC), correlation coefficient of calibration (r). The r values of all six calibration models were determined to be greater than 0.94, suggesting each model is reliable. Therefore, the quantitative NIR models reported in this study can be qualified to accurately quantify the contents of six medicinal components in *C. tubulosa*.

## 1. Introduction

*Cistanche* (Hoffmg. Et Link) is a perennial phanerogamic genera of the Orobanchaceae family of plants. Most species belonging to *Cistanche* genus have been used as a medicinal plants for millennia in China; have a reputation as a superior tonic; and are known as “Ginseng of the Deserts” [1,2]. *Cistanche tubulosa* is an obligate parasite of the roots of perennial plant *Tamarix chinensis*. It has been documented in the Chinese Pharmacopoeia as the authentic source of Cistanches Herba (Chinese name: Roucongrong) from 2005 edition [3]. Modern pharmacological researches on *Cistanche* species initiated at the 1980s [4]. Pharmacological investigations showed that the extracts of *Cistanche* plants possess a wide spectrum of activities, such as curing kidney deficiency and senile constipation, advancing the ability to learn and memorize, anti-Alzheimer’s disease, enhancing immunity, anti-aging, anti-fatigue, etc. [1,5,6,7]. In the last three decades, comprehensive and systematic pharmacological studies have been combined with phytochemical investigations to illuminate the material basis of the beneficial effects of the roots of *Cistanche* plants. These surveys indicate that phenylethanoid glycosides (PhGs) were the main effective components in *Cistanche* plants playing key roles for the treatment of kidney deficiency, impotence [8], anti-aging [9] and anti-Alzheimer’s disease [10]. The contents of two PhGs (echinacoside and verbascoside) were required in Chinese Pharmacopoeia. Meanwhile, carbohydrates such as mannitol, sucrose, glucose and fructose in *Cistanche* plants own the laxative function and the carbohydrate clusters of Cistanche plants have been used for the treatment of constipation [11].

Wild resources of *C. tubulosa* are mainly distributed in the area surrounding the Taklamakan Desert in the southern Xinjiang Autonomous Region in China. Similar to many other species used as traditional Chinese medicines (TCMs), *C. tubulosa* is of great economic value and almost extinct in its wild habitat because of over-collection. Cultivation of *C. tubulosa* began in the 1990s in China to ensure the supply of raw materials for Cistanches Herba as well as protect wild plant resources. As of 2017, nearly 13 thousand ha of cultivated *C. tubulosa* exist in Hotan Prefecture in Xinjiang [12,13]. Advances in planting technology are demanded for expanding the cultivation as well as improving the quality of *C. tubulosa*.

The primary purpose of cultivating *C. tubulosa* is to produce Cistanches Herba, being rich of those effective components. However, the contents of the effective components in Cistanches Herba, such as PhGs and oligosaccharides, can be significantly affected by many factors during production [12,13]. A real-time detection system of quality of *C. tubulosa* should be explored. Therefore, it is necessary to develop a high throughput method to fulfill the requirement of analyzing a large number of samples within a short period of time. Traditionally, the determination of those primary effective components, such as PhGs and carbohydrates, in *C. tubulosa* was usually achieved using high performance liquid chromatography (HPLC) [14,15]. Although being accurate and reliable, it is time-consuming and laborious for data collection and processing. In addition, a great deal of time and efforts are also required for sample preparation that usually involves pulverization, extraction, and filtration, of HPLC assays. Therefore, a clear principle and easy-to-operate tool are needed to obtain a relatively large amount of data. Fortunately, near infrared spectroscopy (NIRS) has been extensively used to assess agricultural products [16], food [17], medical samples [18], and pharmaceutical products [19] because it is rapid as well as nondestructive. Therefore, NIRS could exactly matching the requirements for efficient measurements of TCMs, and it is unsurprising that NIRS has been applied for qualitative identification [20,21] and the quantification of compounds [22] in TCMs.

In this study, the contents of six effective components, including echinacoside, verbascoside, mannitol, sucrose, glucose and fructose in 116 batches of *C. tubulosa* samples that were collected from Hotan Prefecture in Xinjiang among 2013–2015 were firstly determined by HPLC. Afterwards, the calibration models of these six components were established with the partial least squares regression (PLSR) method. These models were then validated with the correlation coefficients and prediction errors in the calibration sets. The results demonstrated that the developed method could be employed as a reliable method for quantitative analysis of *C. tubulosa*.

## 2. Results

### 2.1. HPLC Analysis

**Figure 1:**
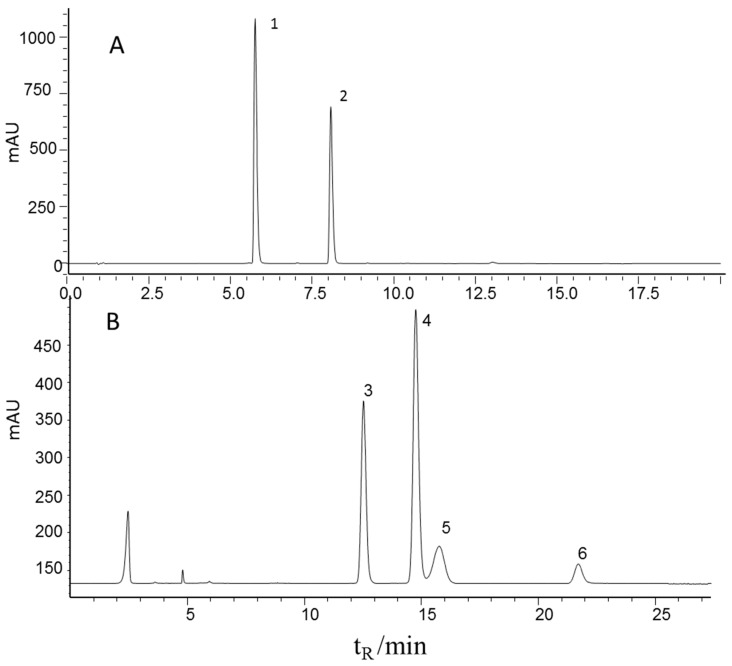
Representative chromatograms of the mixed standards using HPLC-UV (**upper**) and HPLC-ELSD (**lower**) 1, echinacoside; 2, verbascoside; 3, fructose; 4, mannitol; 5, glucose, 6, sucrose.

The contents of echinacoside and verbascoside were determined by a well-defined HPLC-UV method in the literature [3,23] and four carbohydrates (mannitol, sucrose, glucose, and fructose) were determined by a well-defined HPLC-ELSD method in the literature [24] for all the 116 samples. Sample preparation and determination methods were described in Section 3.1 and Section 3.3. Figure 1 shows the characteristic chromatograms of the mixed standards. It can be seen that all six effective components were baseline separated and therefore could be quantified. The HPLC method was validated before the sample testing. The main results of the HPLC method are listed in Table 1. A favorable linear relation (r = 0.9998) and recovery (98.5%) of the echinacoside determination method are shown in the results, the same result as all the five components. Therefore, the contents of the six effective components can be determine accurately. All determined content ranges are summarized in Table 1.

### 2.2. NIRS Analysis

**Figure 2:**
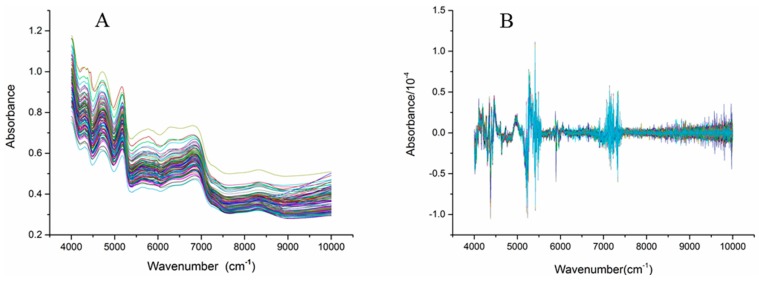
Near Infrared spectra of the *C. tubulosa* samples (**A**) and the spectra processed with 2nd derivation (**B**) (n = 116)..

Figure 2 shows the NIR spectra (4000–10,000 cm^−1^) of the *C. tubulosa* samples. Significant absorption peaks appeared from 4000 cm^−1^ to 7500 cm^−1^ in all samples, while gentle fluctuations appeared from 7500 cm^−1^ to 10,000 cm^−1^. Baseline drift of the NIR spectra occurred because the sample was easily affected by factors such as particle size and color (Figure 2A). Mathematical pretreatments of the spectra were used to reduce the influence of unnecessary information to some degree. The mathematical pretreatments included first derivation (1st derivation), second derivation (2nd derivation), standard normal variety (SNV) and multiplicative scatter correction (MSC). Figure 2B shows the 2nd derivation of the NIR spectra of *C. tubulosa*, and the significant variations occurred from three regions, 4000–4500 cm^−1^, 5000–5500 cm^−1^, and 7000–7500 cm^−1^, are obviously observed.

### 2.3. Establishment of Quantitative Calibration Models

Partial least squares regression (PLSR) is a classic modeling method and it has been widely applied in quantitative models because of the high quality of the results. The advantages of PLSR include its good forecasting ability and relatively simplicity. PLSR has also widely applied in the establishment of quantitative calibration models of TCMs [25]. Based on the pretreated NIR spectra, an NIR quantitative analysis model for the six effective components in *C. tubulosa* was established using the PLSR method with HPLC analysis data as the true values. The 116 samples were randomly divided into calibration and validation sets with a 3:1 ratio. The most suitable conditions for the calibration were chosen by low RMSEC and high correlation coefficient.

#### 2.3.1. Selection of the Wave Band for the Calibration Models

The selection of suitable wave band was an important step for calibration models building. In this study, the NIR interval spectra of 4000–7500 cm^−1^ (recommend by TQ analyst software) and 4000–10,000 cm^−1^ were compared. It was observed that this range unsuitable for the calibration at the interval between 4000 cm^−1^ and 7500 cm^−1^ from Table 2. Hence, in the current study, the spectral intervals for the six chemical constituents were all selected from the interval from 4000 to 10,000 cm^−1^ by comparing the performances of RMSEC and correlation coefficient.

#### 2.3.2. Selection of the Optimum Number of Factors for the Calibration Models

**Figure 3:**
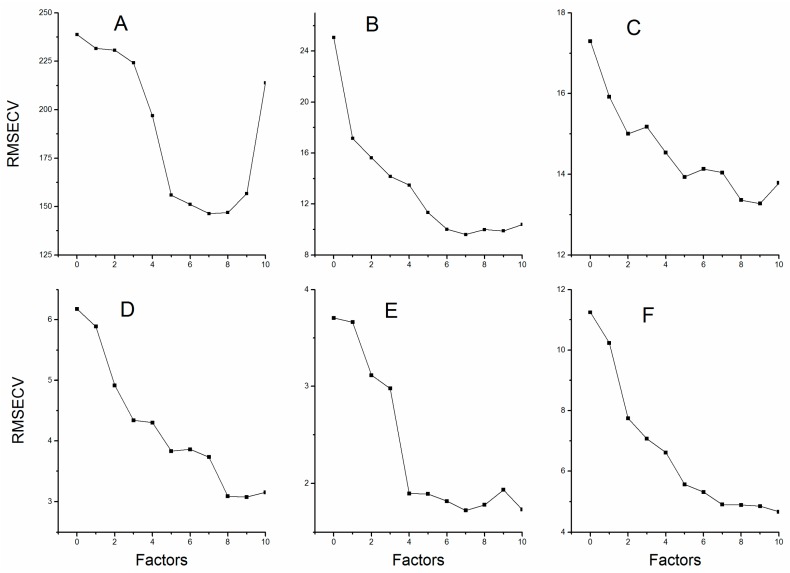
The root mean square errors of cross validation with different number of factors: (**A**) Echinacoside; (**B**) Verbascoside; (**C**) Mannitol; (**D**) Sucrose; (**E**) Glucose; and (**F**) Fructose.

The PLSR explains the maximum amount of variability in the data through reducing the dimensionality of the spectra data by the calculation of factors. The “underfittedness” problem appeared due to insufficient information which resulted from limited number of factors; however, choosing the factors greater than the optimum values introduced in the model will bring about the “overfittedness” problem. Either “underfittedness” or “overfittedness” will reduce the predictive power of the established models [22]. Figure 3 shows the relation between RMSECV and factors for all six compounds. Therefore, we selected those factors corresponding to the lowest values of RMSECV. The optimum selection of factors for the calibration models is listed in Table 3.

#### 2.3.3. Selection of Spectral Pretreatment for the Calibration Models

Another most critical influential factor for calibration models is spectral pretreatment which aimed at reducing the influence of scattering and baseline drift, enhancing signal-to-noise ratios and removing irregular variations. Multiplicative scatter correction (MSC) and standard normal variate (SNV) methods were used to eliminate the influence of radiation scattering customary. To solve the effects of baseline drift, 1st and 2nd derivative spectra were compared and the 2nd derivation was selected [26]. For the desirable effect, we smoothed the spectra with the Savitzky–Golay (SG) filter algorithm before derivation to prevent noise magnification. Table 3 shows the information of spectral pretreatment and its results for the calibration models.

### 2.4. Evaluation of the Established Models

**Figure 4:**
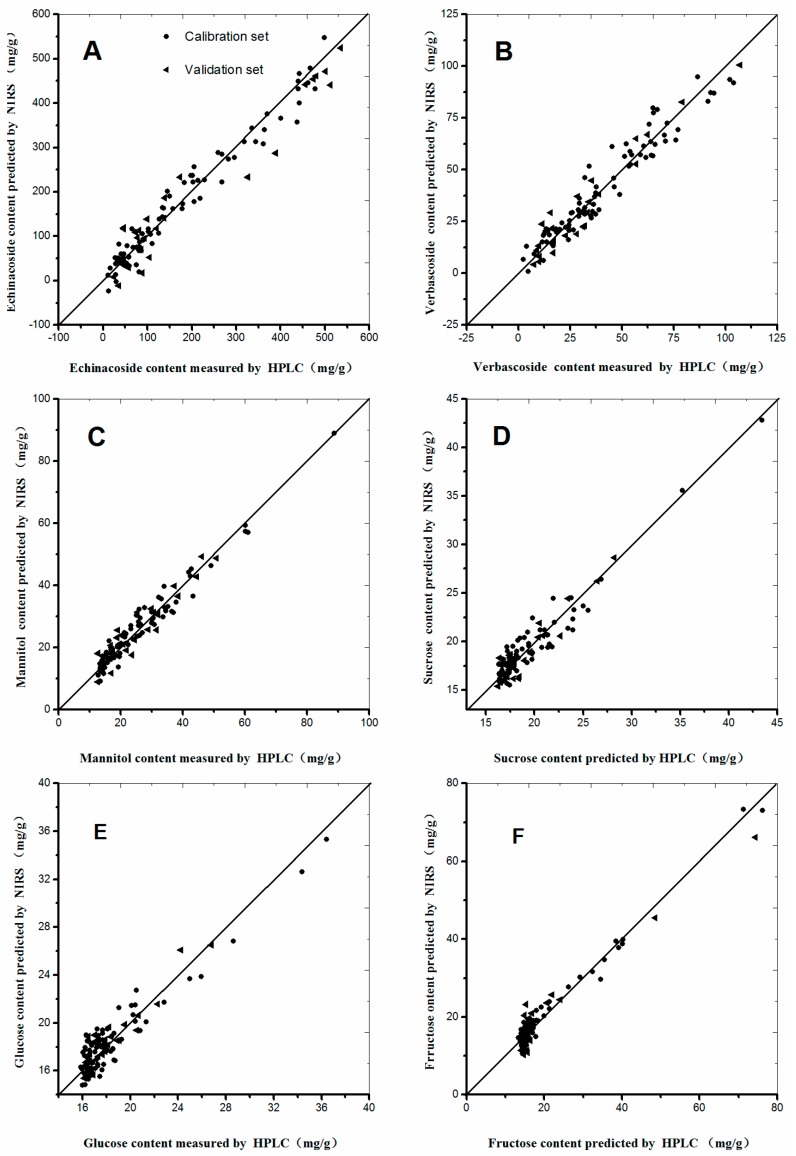
Scatter plots of measured and predicted values for the abundance of six constituents of *C.* *tubulosa* in the calibration and validation sets: (**A**) Echinacoside; (**B**) Verbascoside; (**C**) Mannitol; (**D**) Sucrose; (**E**) Glucose; and (**F**) Fructose.

A good NIRS calibration model should have low RMSEC and RMSEP values, as well as a high correlation coefficient (*r*) and small differences between RMSEC and RMSEP [27,28,29]. The calibration models of the six selected compounds were established according to the procedures mentioned above (Table 3). The RMSEC and *r* values for the calibration set of echinacoside were 27.6 and 0.9808, respectively. The performance parameters of other chemical compound models are listed in Table 3, from which we can concluded that the established models emerges satisfactory prediction results, and can be used for the rapid quantitative analysis of *C. tubulosa*. Scatter plots of the six chemical compounds are shown in Figure 4 to make the calibration models more descriptive and observed visually. As shown in Figure 4, minor differences occurred between the predictive and measured values, because most dots were distributed around the regressive curve with an equation as *y* = *x*. Therefore, excellent predictive performances were observed in Figure 4.

## 3. Materials and Methods

### 3.1. Sample Preparation

One hundred sixteen *C. tubulosa* samples were collected from Hotan Prefecture in Xinjiang autonomous region from 2013 to 2015. All samples were cultivated, but they were collected at different growth stages. The fresh weight of the samples ranged from 20 g to 1000 g. After sun drying, the dried samples were crushed and sifted through a 60-mesh sieve [3,23].

### 3.2. NIR Spectroscopic Data Collection

The NIR spectra of the samples were collected at an 8 cm^−1^ interval over the spectral region of 4000–10,000 cm^−1^ with an Antaris MXFT-NIR System (Thermo Scientific, Madison, WI, USA) equipped with a hand-held optical fiber reflectance adapter. Each spectrum was obtained by averaging 64 scans. All samples were allowed to equilibrate to room temperature (25 °C) before NIR spectra scanning to ensure that the samples were analyzed at the same temperature. The humidity in the laboratory was kept at an ambient level.

### 3.3. HPLC Data Collection

#### 3.3.1. Extraction Preparation

One gram of *C. tubulosa* powder was extracted with 50 mL of 50% methanol in a conical flask with ultra-sonication (500 W, 40 KHz) for 30 min. The extract was stored at 4 °C. The supernatant of the extract was filtered to obtain a sample for HPLC analysis [3,23].

#### 3.3.2. Simultaneous Determination of Echinacoside and Verbascoside with HPLC-UV

Liquid chromatographic analysis was conducted on a Shimadzu UHPLC system (Shimadzu, Kyoto, Japan) consisting of two LC-20ADXR solvent delivery units, a LC-20AD pump, a SIL-20ACXR auto sampler, a CTO-20AC column oven, a SPD-M20A DAD detector, DGU-20A3R degasser, and a CBM-20A controller.

A Grace Prevail Carbohydrate ES column (150 × 2.1 mm, 2.7 mm) used for the chromatographic separations was maintained at 35 °C. The mobile phase consisted of acetonitrile (A) and 0.1% aqueous formic acid (B) and was delivered following the gradient program as follows: 0–7 min, linear gradient of 10–20% A; 7–15 min, 20% A; and 15–20 min, linear gradient of 20–10% A. The flow rate of the mobile phase was 0.4 mL/min. UV-monitoring was performed at 330 nm.

#### 3.3.3. Simultaneous Determination of Mannitol, Sucrose, Glucose and Fructose with HPLC-ELSD

HPLC was performed on an Agilent 1100 series LC system (Palo Alto, CA, USA) consisting of a G1322A degasser, a G1311A quaternary pump, a G1311A auto sampler, a G1316A column temperature controller and a G1315B DAD detector.

A Sigma Prevail Carbohydrate ES column (4.6 × 250 mm, 5 μm) was employed for chromatographic separations and maintained at a column temperature of 25 °C. The mobile phase was composed of acetonitrile and water (77:23, *v*/*v*) and isocratic ally supplied at a flow-rate of 0.7 mL/min. The effluent was monitored using an evaporative light scattering detector (ELSD) with defaulted parameters [23,24].

### 3.4. Data Processing

TQ Analyst (version 8.0, Thermo Scientific, Madison, WI, USA) was used to perform division of the calibration and validation sets, mathematical pretreatment of the spectra, establishment of the calibration models, and other computations. Origin (version 9.1) was used to make the figures.

## 4. Conclusions

In this study, we proposed a rapid and non-destructive method for the simultaneous analysis of echinacoside, verbascoside, mannitol, sucrose, glucose and fructose in *C. tubulosa* by NIRS. Analyses of RMSEC, correlation coefficient, RMSEP and Rp values demonstrated that the established quantitative NIR models could be used to accurately predict the contents of the six selected effective components in *C. tubulosa*. In comparison with HPLC, the NIRS method reported in this study can save significant labor and time, while maintaining satisfactory quantitative analysis ability. Therefore, the method reported here has the potential to be used in the quality control of *C. tubulosa* and thus to guide the development of cultivation and process technology for *C. tubulosa*.

## Figures and Tables

**Table 1:** Main results of the HPLC method.

Medicinal Components	*t*_R_ (min)	Standard Sample Range (mg/g)	Calibration Curve	r	Recovery (%, *n* = 3)	Sample Value Range (mg/g)
Echinacoside	5.79	1.00–1000.00	*Y* = 1.106 × 10^−4^*X* + 2.50	0.9998	98.5	6.50–535.45
Verbascoside	8.10	1.00–250.00	*Y* = 8.100 × 10^−5^*X* + 0.03	0.9995	103.8	1.35–212.88
Fructose	12.50	1.00–250.00	*Y* = 4.195 × 10^−2^*X* + 2.13	0.9999	100.5	13.36–104.78
Mannitol	14.70	1.00–100.00	*Y* = 4.954 × 10^−3^*X* + 0.113	0.9990	99.6	12.52–88.70
Glucose	15.70	1.00–100.00	*Y* = 1.857 × 10^−2^*X* + 0.104	0.9999	100.2	15.84–75.36
Sucrose	21.60	1.00–100.00	*Y* = 6.816 × 10^−3^*X* + 0.156	0.9996	98.5	16.22–43.43

**Table 2:** Performance comparison of different wave band choose of calibration models.

Medicinal Components	Performances of RMSEC and *r*	Wave Band	
		4000–10,000 cm^−1^	4000–7500 cm^−1^
Echinacoside	RMSEC	27.9	136.0
	*r*	0.9813	0.3431
Verbascoside	RMSEC	6.69	11.00
	r	0.9629	0.8956
Mannitol	RMSEC	3.05	12.5
	*r*	0.9747	0.4029
Sucrose	RMSEC	1.21	2.63
	*r*	0.9584	0.786
Glucose	RMSEC	1.22	2.16
	*r*	0.9426	0.8074
Fructose	RMSEC	1.57	6.75
	*r*	0.99	0.7951

**Table 3:** Most suitable pretreatment and performance comparison of results for the calibration models.

Medicinal Components	Optimum NO. of Factors	Pretreatment Spectral	Calibration Set		Validation Set	
			RMSEC	*r*	RMSEP	Rp
Echinacoside	7	MSV, 1st D, SG (7, 3)	44.00	0.9531	42.70	0.9601
		MSV, 2nd D, SG (7, 3)	27.60	0.9808	24.53	0.9688
Verbascoside	7	SNV, 1st D, SG (15, 3)	6.84	0.9612	7.28	0.9637
		SNV, 2nd D, SG (15, 3)	6.76	0.9627	6.57	0.9617
mannitol	9	SNV, 1st D, SG (7, 3)	10.50	0.6350	14.00	0.1287
		SNV, 2nd D, SG (7, 3)	2.85	0.9775	3.32	0.9460
Sucrose	9	MSV, 1st D, SG (7, 3)	1.63	0.9228	1.34	0.9456
		MSV, 2nd D, SG (7, 3)	1.19	0.9597	1.02	0.9449
Glucose	7	SNV, 1st D, SG (17, 3)	1.21	0.9437	1.09	0.9006
		SNV, 2nd D, SG (17, 3)	1.20	0.9441	1.00	0.9336
Fructose	10	SNV, 1st D, SG (13, 3)	3.70	0.9432	4.75	0.9252
		SNV, 2nd D, SG (13, 3)	1.55	0.9902	1.74	0.9689
